# Subtle impairments of facial emotion expressions in individuals at ultra-high risk for psychosis

**DOI:** 10.3389/fpsyt.2026.1743609

**Published:** 2026-04-01

**Authors:** Tina D. Kristensen, Bjørn H. Ebdrup, Karen S. Ambrosen, Merete Nordentoft, Louise B. Glenthøj, Stella Grasshof

**Affiliations:** 1 Center for Neuropsychiatric Schizophrenia Research (CNSR), Mental Health Center Glostrup, Copenhagen University Hospital – Amager and Hvidovre, Hvidovre, Denmark; 2 Department of Clinical Medicine, Faculty of Health and Medical Sciences, University of Copenhagen, Copenhagen, Denmark; 3 Department of Psychology, University of Copenhagen, Copenhagen, Denmark; 4 Copenhagen Research Center for Mental Health (CORE), Mental Health Center Copenhagen, Hellerup, Denmark; 5 VIRTU Research Group, Mental Health Center Copenhagen, Hellerup, Denmark; 6 Data Science Section, IT University of Copenhagen, Copenhagen, Denmark

**Keywords:** Action units (AUs), automated registration, emotion expression, facial features, ultra-high risk for psychosis

## Abstract

Emotional processing deficits are increasingly recognized as a salient feature of psychotic disorders, and emerging evidence suggests that impairments in facial emotion processing may serve as additive predictive markers for individuals at ultra-high risk (UHR) for psychosis, highlighting the importance of such deficits for early identification and intervention strategies. In this study we examined whether automated coding of facial features from brief video recordings (45 seconds) could discriminate 108 individuals at ultra-high risk for psychosis (UHR) from 65 matched healthy controls (HC). We assessed the alignment between automated coding of facial expressivity as measured by granulated muscle activity, and clinician-rated facial emotional expressivity by experienced clinicians. In addition, we explored associations with social skills and clinical symptoms. A random forest classifier with repeated nested cross-validation significantly classified UHR-individuals from HC with above chance accuracy (63%; *p* = 0.009, sensitivity=0.76, specificity=0.40). Automated coding of Presence, Intensity, and Variation of specific facial features correlated strongly with clinician-rated facial expressivity with moderate to large effect sizes (all corrected *p* < 0.001). Moreover, facial feature metrics were associated with attenuated psychotic symptoms and negative symptoms, supporting the clinical validity of automated facial expressivity coding from brief video recordings of UHR-individuals.

## 1 Introduction

Individuals at ultra-high risk (UHR) for developing psychosis are clinically identified by attenuated psychotic symptoms (APS), brief limited or intermittent psychotic symptoms, or state-and-trait vulnerability (a first-degree relative with a psychotic disorder or meeting criteria for schizotypal personality disorder). These features are commonly assessed using standardized instruments such as the Structured Interview for Psychosis-Risk Syndromes (SIPS) (1) or the Comprehensive Assessment of At-Risk Mental States (CAARMS) (2). The UHR construct represents a clinically important preventive model for identifying individuals at elevated risk for psychosis. However, given that only approximately 10–30% of identified UHR-individuals transition to a psychotic disorder within three years (3), there remains a pressing need for supplementary clinical tools capable of distinguishing those at true, imminent risk, who may benefit from more intensive interventions (4). Importantly, even among individuals who do not transition, persistent social and functional impairments (5) linked to broader social cognitive difficulties (6) highlight the need for feasible, objective assessment methods to identify clinically meaningful markers and potentially modifiable intervention targets at this early stage (7).

Emotion processing is a core component of social cognition implicated across the psychosis spectrum, and impairments in this domain have been proposed as early indicators of psychosis risk (8). Within emotion processing, facial emotional expressivity is central for effective interpersonal communication and social reciprocity and is essential for understanding the nuances of emotional processing impairments. UHR samples typically demonstrate moderate deficits in facial affect recognition and discrimination relative to healthy controls (9). In established psychotic disorders, blunted facial affect, manifested as reduced intensity, frequency, and variability of expressions, is a well-documented symptom across both clinician-rated and computational assessments (10). Nevertheless, the clinical relevance and phenotype of facial emotional expressions in UHR populations remain insufficiently understood (11).

A widely used framework for quantifying facial expressions is the Facial Action Coding System (FACS), which decomposes complex facial expressions into anatomically based muscle movements known as Action Units (AUs) (12). Manual FACS coding shows high consistency with clinical rating approaches, indicating its potential utility in clinical assessment (13). However, manual coding is time- and resource-intensive, depending heavily on trained human coders, which limits the feasibility in routine clinical practice. Consequently, automated facial analysis software has emerged as potentially objective and time-efficient alternatives for assessing emotion processing deficits. One key advantage of automated systems is their ability to analyze facial expressions in real time, using fine-grained and standardized metrics that may capture subtle abnormalities characteristic of UHR individuals overlooked by conventional rating (14) and provide a more dynamic understanding of emotional responses during social interactions (15).

Automated FACS-based toolkits [e.g., OpenFace (16)] generate itemized, time-resolved AU metrics which are scalable, rater-independent, and suitable for brief, naturalistic interviews. However, because most automated emotion recognition systems have been trained and validated on static, standardized facial expression datasets (17, 18), there is a critical need for validation studies in clinical UHR populations (19). In schizophrenia research, deep learning approaches applied to videos of micro expressions have achieved very high classification accuracy (20), and neural network models trained on facial expression databases have yielded comparable performance (21). Clinical relevance has also been demonstrated in severe mental illness, where clinician-rated blunted affect was associated with automated estimates of higher-level emotions such as anger, fear, and sadness (22). In UHR/clinical high-risk (CHR) youth, Gupta et al. have reported abnormalities in higher-level emotional expressivity, such as blunted joy, which were associated with increased psychosis conversion risk (14, 23). Notably, Gupta et al. also demonstrated convergence between automated and human ratings of emotional expressivity based on combinations of multiple AUs, but emphasized the need for further validation at the level of individual AUs and careful comparison with manual coding approaches due to observed discrepancies (24). As facial expression analysis is advancing in mental health, there is an increasing need for systematic, concurrent validation of automated AU features in larger UHR/CHR samples, particularly studies examining alignment with clinician-rated expressivity and associations with clinical outcomes.

In this study, we evaluate whether machine learning models based on automated AU coding can (1) distinguish UHR individuals from healthy controls using granular, anatomically defined AUs, and (2) we investigate the clinical validity by assessing whether automated AU outputs are associated with (a) clinician-rated facial expressivity (primary outcome), and (b) social skills, attenuated psychotic symptoms, and negative symptoms (exploratory outcomes). By positioning automated AU analysis not only as a classification method but as a potentially clinically validated tool for assessing emotion processing deficits in UHR-individuals, this study aims to bridge the gap between computational promise and clinical utility.

## 2 Methods

The study followed the Declaration of Helsinki and was approved by the Capital Region of Denmark’s Ethics Committee (H-6-2013-015) and the Danish Data Protection Agency (2007-58-0015). All participants provided informed oral and written consent.

### 2.1 Participants

We included 108 help-seeking UHR-individuals (aged 18-40) from the Mental Health Centre Copenhagen, Denmark, as part of the FOCUS-trial (25). UHR-status were identified using the Comprehensive Assessment of At-Risk Mental State (CAARMS) (26), fulfilling the criteria of sufficient intensity and frequency of attenuated psychotic symptom; brief limited intermittent psychotic symptoms; or trait (schizotypal personality disorder) and/or genetic vulnerability (1^st^ degree relative), along with a significant drop in functioning or sustained low functioning through the past year. Current or lifetime psychotropic medication were accepted, including antipsychotics. Exclusion criteria were history of a psychotic episode of more than one week duration; psychiatric symptoms explainable by a physical illness with psychotropic effect or acute intoxication (such as cannabis use); a diagnosis of a serious developmental disorder (e.g., Asperger’s syndrome or IQ<70); or currently receiving methylphenidate. Sixty-five HC were concurrently included and matched to the UHR-individuals on age, gender, and parental socioeconomic status. HC were recruited through internet and community-based advertising, and had no current or previous psychiatric diagnoses, substance abuse or dependency as established using the Structured Clinical Interview for DSM-IV Axis I and Axis II disorders (SCID) (27), and no first-degree relative with a psychotic disorder. The SCID assessors were all certified in SCID diagnostic interviewing. While prior studies used overlapping samples (28), this study only included a subsample with never analyzed baseline data from FACS.

### 2.2 Assessments

Diagnostic assessment included CAARMS, SCID-I and parts of SCID-II (29). CAARMS composite score measured the level of attenuated psychotic symptoms; and the SANS (30) assessed negative symptoms. Social skills were evaluated using the High-Risk Social Challenge (HiSoC) (31), a standardized, performance-based social skills test with proven high validity and reliability in adolescents at genetic high risk for psychosis (32). It is a videotaped task, in which the participants are instructed to do a 45-second audition on being the most interesting person in the country, as a mock competition with a grand money prize. Standardized test instruction is read aloud to the participant, which is given 10 seconds preparation. The interviewer records the “audition” but do not intervene or support with further questions to the participant. The scoring of the task comprises 16 items, such as Item 1. Facial affect: *The degree, amount, range, and naturalness of emotions displayed via facial expression*, or Item 5. Gaze: *Frequency, duration, appropriateness, and naturalness of eye contact*, see **Supplementary Table 1** for all items. Experienced psychologists conducted the scoring of the HiSoC based on the manualized scoring procedure of the videotaped task using consensus scoring, with Items rated on a five-point Likert scale (higher scores indicating better social skills). Video sequences were recorded frontally per protocol. Furthermore, three factors based on the original factor analysis of the task (32): “Affect”, “Odd Behavior and Language”, and “Social-Interpersonal”, along with a general impression of the performance is rated. The “Affect” factor includes above Item 1. also non-verbal expressions, gestures, appropriateness of affect, verbal expression, gaze and physical anergia. The “Odd Behavior and Language” factor includes appearance, unusual or odd behavior, inappropriate dressing, awkward ticks or jerks, but also verbal content as unusual or odd speech, irrelevant answer, unclarity, neologisms, word salat, tangentiality, derailment, loose associations, and valence. Finally, the “Social-Interpersonal” factor includes fluency of speech, guardedness, social anxiety, and engagement in task. The three factors and the rater’s general impression of social skills are added to compute a HiSoC total score, reflecting overall social performance skills. Interrater reliability was high (ICC = 0.88-0.98) (33). The primary outcome chosen for clinical validation analyses of the automated coding of AU was the HiSoC Item 1. Facial affect: *The degree, amount, range, and naturalness of emotions displayed via facial expression*. This item most directly reflects the anatomical facial expressions, with low ratings when the facial affect appears restricted, with dull, flat, or blank expressions and no affect displayed throughout the video; across some variation in smiling, blinking, frowning, lifting eyebrows etc.; to the highest scores when the facial expressions unrestricted enhances the verbal communication, without being forced.

### 2.3 Facial features

**Figure 1:**
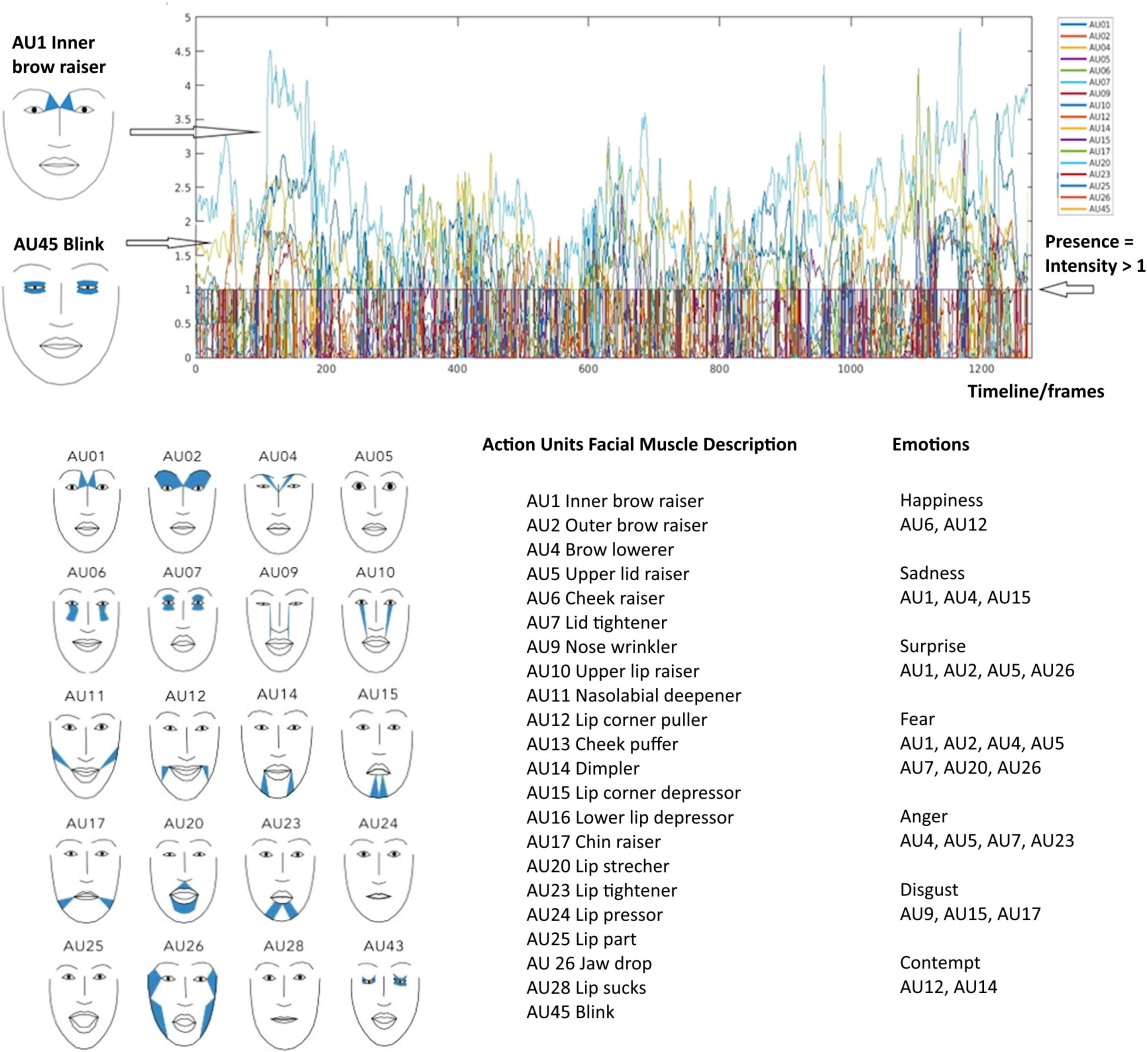
Definition action units. Top panel illustrates the action units (AU) metrics of Presence and Intensity on a timeline. The 18 AUs are represented by different colors defined at the panel to the right. Presence indicates whether a certain AU is detected above threshold of intensity 1 in the given time frame. Intensity indicates how intense an AU is on a scale of 0–5 in a given time frame. Bottom panel left show the visualizations of AUs as defined by PyFeat (Cheong JH, Jolly E, Xie T, Byrne S, Kenney M, Chang LJ. Py- feat: python facial expression analysis toolbox. Affect Sci. 2023; 4(4):781-796. doi:10.1007/s42761-023-00191-4). Bottom panel right defines the AUs at the level of facial muscle activation, as well as defines which AUs is implicated in expression of higher level emotions.

HiSoC videos were analyzed using OpenFace 2.0 to extract 18 AUs based on the Facial Action Coding System (FACS) framework (16). **Figure 1** contains the complete list of the 18 AUs, including visualizations from PyFeat. For each frame and AU, OpenFace estimates if AU is active as “*Presence*” (binary: 0 or 1) and “*Intensity*” (continuous, range 0-5). Frames with a confidence level <80% were removed; participants with <90% valid frames were excluded.

As quality control, we visually inspected all videos and did not observe noticeable or consistent downward tilts, or differences in head pose. We did however remove cases of low confidence. To further evaluate the potential nuisance impact of head pose we computed the correlation between pitch, yaw, and roll and all AUs. As values were very small, we concluded that effects were negligible (**Supplementary Table 2**).

Because sequence lengths differed across recordings, features independent of duration were computed. For Presence and Intensity, the mean and standard deviation were calculated. In addition, the proportion of frames with intensity values >1, and the proportion of switches between active and inactive presence relative to sequence length [%] as “*Variation*”. This yields six features per AU per sequence which were used as input for the binary classifier.

For the correlation analysis we selected three AU features: *Presence* (proportion of frames with intensity>1), *Intensity* (mean intensity on the 0–5 scale), and *Variation* (activation flips relative to signal length), see **Figure 1** for illustration.

### 2.4 Statistical analysis

**Figure 2:**
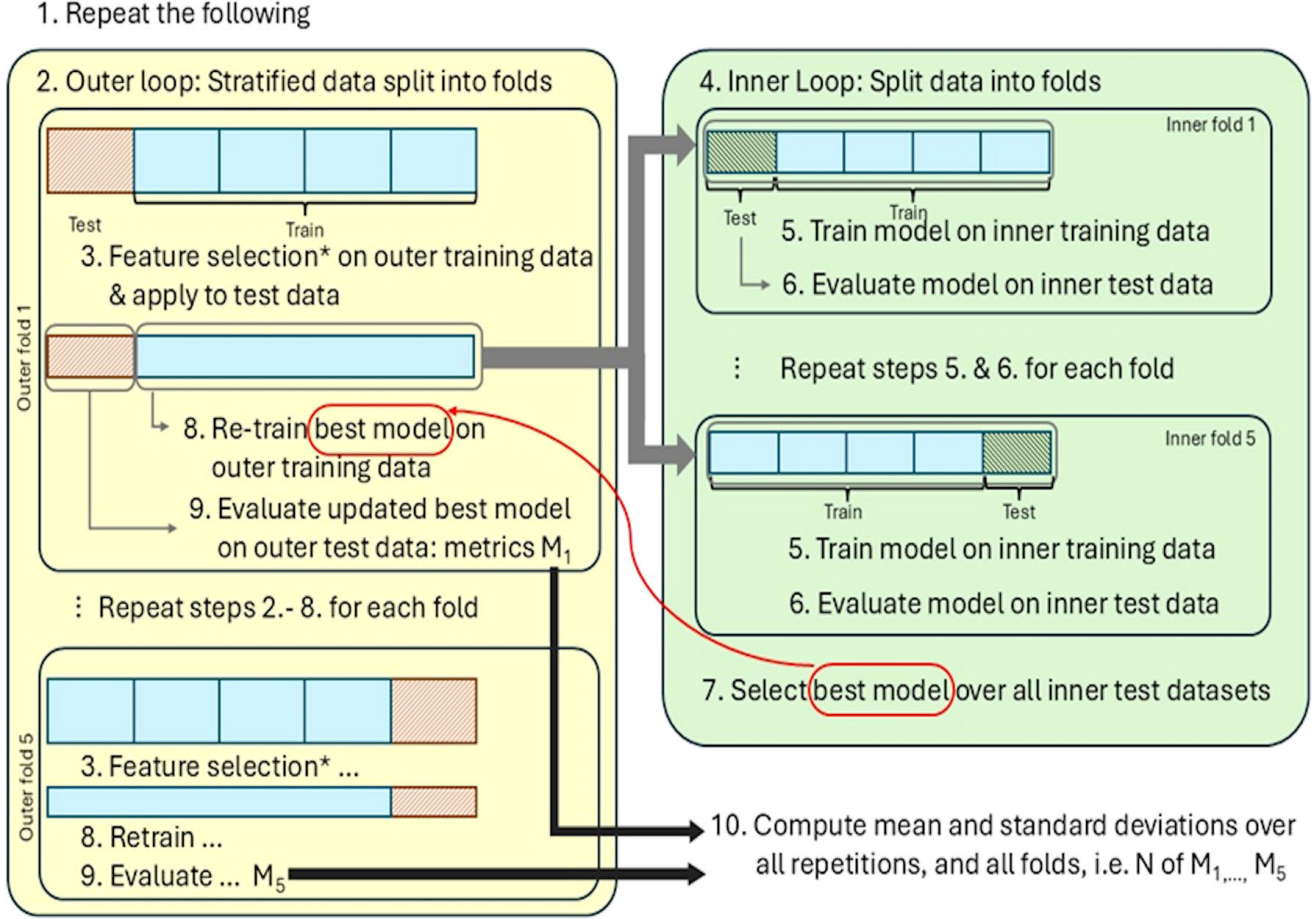
Overview of the repeated nested cross-validation pipeline. Illustration of the process for repeated nested stratified cross-validation. For the permutation testing, we permuted the labels before feature selection (step 3.), indicated by the star (*). The pipeline is described in detail in **Supplementary Text S1**.

Demographic and clinical data were analyzed using univariate tests in SPSS v24.0. A Random Forest classifier was used to distinguish UHR-individuals from HC based on AU metrics. Random Forest was selected because it performs well on small, high-dimensional datasets, is robust to multicollinearity, and captures non-linear relationships (34). To evaluate the robustness of our findings among different model families, we additionally implemented a SVM classifier. Model performance was evaluated using repeated nested stratified cross-validation with permutation testing. This approach was chosen to mitigate overfitting, optimize hyperparameters, and assess statistical significance. Stratification preserves class proportions across folds, and repetition of cross-validation improves stability of performance estimates in small samples. The procedure is illustrated in **Figure 2**.

In each outer loop, the dataset was stratified and split into training and test sets. Feature selection was performed exclusively on the training data to prevent information leakage from the test set. If no features were selected, all features were retained by default. (This never occurred with the real data, only with permuted labels). The model was then trained on the full training set and evaluated on the held-out test set. To address potential class imbalance, we selected the best-performing model per fold based on the Area Under the Curve (AUC) and reported the mean performance metrics (accuracy and AUC) across all outer folds and repetitions.

Within each outer fold, an inner loop was used for hyperparameter tuning. The training data was further split into stratified inner folds, where models were trained and validated to identify the optimal hyperparameters. The final model was retrained on the entire outer training set using the best hyperparameters before evaluation on the outer test set.

To address group imbalance, the best AUC per fold was selected, and performance metrics were averaged across 5 outer, 5 inner folds and 5 repetitions. The whole procedure was repeated with the permuted labels in both outer and inner loops 100 times for significance testing, yielding p-values. See **Supplementary Text S1** for details.

Secondary analyses included Pearson’s correlations tests between Presence, Intensity, and Variation of 18 AU metrics and HiSoC item 1 as the primary outcome (Facial affect: The degree, amount, range, and naturalness of emotions displayed via *facial* expression) (16). Main analyses were done on all participants, *post hoc* analyses pertained to group specific tests. To account for multiple comparisons in main analyses, we corrected using both false discovery rate, as well as Bonferroni correction (significance level at (0.05/53 tests) p<0.0009).

Exploratory analyses examined the associations between Presence, Intensity, and Variation of 18 AU metrics and HiSoC factors/total scores, as well as clinical symptoms (CAARMS and SANS) in UHR-individuals using Pearsons’s correlation test, with significance level corrected for multiple comparisons using false discovery rate (FDR).

*Post Hoc* sensitivity tests further exploring potential medication effects, were performed comparing antipsychotic naive (AP-naive) UHR-individuals with UHR-individuals that had received antipsychotic medication (current or lifetime AP-exposed UHR-individuals) on the Presence, Intensity, and Variation of the 18 AU variables. In case of significant differences, we further compared the HC to the two UHR-subgroups, respectively. Finally, we explored potential effect of sex on the AU group differences.

## 3 Results

We included 108 UHR-individuals, and 65 HC matched on age and sex (**Table 1**). Significant group differences were found in functioning, UHR-symptoms, and social skills (HiSoC), all *p* < 0.001. Antipsychotic-naive UHR-individuals did not differ from AP-exposed on any demographic or clinical measures, see **Supplementary Figure S1**, **Supplementary Table S3**.

**Table 1:** Sociodemographic and clinical data.

Variable Mean (S.D.)/percent (N)	UHR (N = 108)	HC (N = 65)	Significance group effect
**Age**	23.9 (4.4)	23.4 (4.2)	*p* = 0.44, F = 0.59
Sex			
Female	53.3% (57)	58.5% (38)	*p* = 0.307
Clinical			
Functioning (SOFAS)	**56.40 (10.90)**	**88.28 (5.69)**	***p* < 0.001, F = 476.38**
UHR-symptoms (CAARMS)	50.08 (14.31)	1.46 (2.69)	***p* < 0.001, F = 733.36**
Negative symptoms (SANS)	1.43 (0.5)	–	N/A
**Hisoc Total score (social skills)**	**51.45 (7.03)**	**63.23 (6.30)**	***p* < 0.001, F = 122.33**
Hisoc item 1 (facial affect)	**3.00 (0.80)**	**3.86 (0.68)**	***p* < 0.001, F = 52.75**
Hisoc factor 1	**19.12 (3.14)**	**24.03 (2.67)**	***p* < 0.001, F = 111.22**
Hisoc factor 2	**16.66 (2.42)**	**19.85 (2.34)**	***p* < 0.001, F = 72.29**
Hisoc factor 3	**12.85 (2.64)**	**15.41 (2.33)**	***p* < 0.001, F = 41.90**
Hisoc factor 4	**2.71 (0.68)**	**3.88 (0.69)**	***p* < 0.001, F = 120.13**
DSM Diagnosis			
Affective	55.9% (57)	–	
Anxiety	51.0% (52)	–	
Personality disorders	36.3% (37)	–	
Other	17.6% (18)	–	
Medication			
AP naive	65.7% (69)	–	–
Antipsychotics current	31.4% (32)	–	
Antidepressants current	24.5% (25)	–	–
Mood stabilizers current	5.9% (6)	–	–
Benzodiazepines current	7.8% (8)	–	–
Substance use %			
**Nicotine**			***p* < 0.001**
Never/once/monthly/ weekly/daily use	42%/4%/6%/7%/41%	80%/0%/7%/9%/4%	
**Alcohol**			*p* = 0.695
Never/once/monthly/ weekly/daily use	8%/14%/40%/34%/4%	5%/7%/43%/41%/4%	
**Cannabis**			*p=*0.673
Never/once/monthly/ weekly/daily use	69%/15%/9%/6%/2%	64%/20%/7%/9%/0%	

Significant effect of group is marked in bold. AP, antipsychotics; CAARMS, Comprehensive Assessment of At-Risk Mental States; HC, healthy controls; HiSoC, High-Risk Social Challenge task; N, number; SANS, Scale for the Assessment of Negative Symptoms; SD, standard deviation; SOFAS, social and occupational functioning assessment scale; UHR, individuals at ultra-high risk for psychosis.

**Figure 3:**
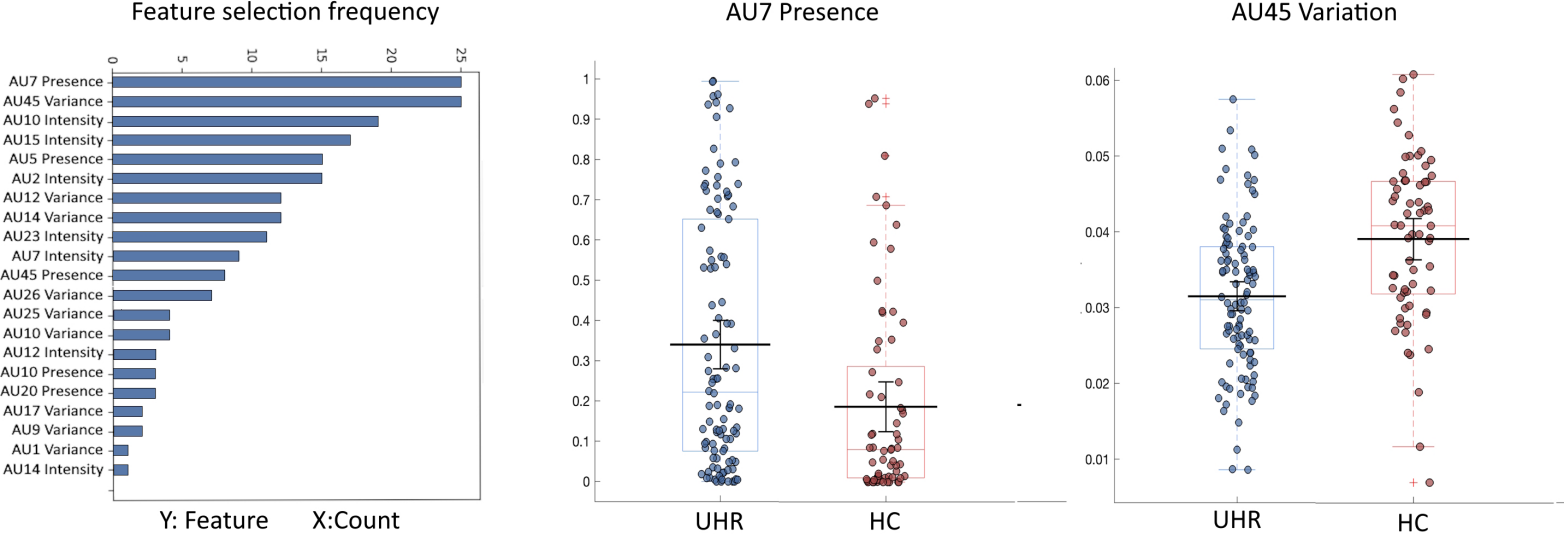
Action units group differences. Left panel visualizes the summed frequency of selection of the 18 AU features of Presence, Intensity, and Variation selected by the random forest classifier. Right panel shows boxplots of the groupwise distribution of the two features Presence AU7 and Variation AU45 selected through all folds and with Bonferroni corrected significant group difference. AU, Action Units; HC, healthy controls; UHR, Individuals at ultra-high risk for psychosis.

Using AU features, the random forest classifier significantly distinguished UHR-individuals from HC (mean accuracy of 0.63 (SD = 0.07), *p* = 0.009, AUC of 0.61 (SD = 0.11), sensitivity of 0.76 (SD = 0.09), specificity of 0.40 (SD = 0.12), and F1 score of 0.72 (SD = 0.06). The frequency of AU features selected is illustrated in **Figure 3** with Presence AU7 (lid tightener) and Variation AU45 (blink) consistently selected throughout all folds. An SVM classifier yielded similar results (mean accuracy 0.62 (SD = 0.08), *p* = 0.009, AUC of 0.62 (SD = 0.10), sensitivity of 0.60 (SD = 0.26), specificity of 0.54 (SD = 0.26), and F1 score of 0.60 (SD = 0.24).

Univariate *post hoc* t-tests showed group differences in multiple AUs (**Supplementary Figure S2**, **Supplementary Table S4**). After FDR correction, UHR-individuals showed more Presence of AU7 (Lid tightener, *corrected p* = 0.014), while HC had more Variation of AU45 (Blink, *corrected p* = 0.002), **Figure 3**. Results were not affected by including sex or antipsychotic medication as covariates, see **Supplementary Tables S5**, **S6**.

Presence, Intensity, and Variation of a range of AUs correlated positively with clinician-rated facial affect expressions (HiSoC Item 1) with effect sizes *r* between 0.21>0.45. After Bonferroni correction for multiple comparisons multiple correlations remained significant, see **Table 2** for specifications. *Post hoc* sensitivity tests revealed that the strongest effect was within the UHR-individuals, see **Supplementary Table S7** for details.

**Table 2:** Association between HiSoC Item 1 and Action Units in all participants.

	Presence	Intensity	Variability
AU1	***r=0.321 p=0.000018 q=0.000150***	***r=0.448 p<0.000001 q<0.000001***	*r=0.151 p=0.048749 q=0.208679*
AU2	***r=0.272 p=0.000328 q=0.001202***	***r=0.301 p=0.000064 q=0.000945***	*r=0.106 p=0.168400 q=0.226953*
AU4	*r=-0.007 p=0.926643 q=0.926643*	*r=-0.021 p=0.780782 q=0.780782*	*r=0.114 p=0.139119 q=0.011262*
AU5	*r=-0.151 p=0.048152 q=0.072228*	*r=0.114 p=0.137163 q=0.332226*	*r=0.104 p=0.176519 q=0.332226*
AU6	***r=0.216 p=0.004630 q=0.010418***	***r=0.210 p=0.005899 q=0.002506***	*r=0.211 p=0.005631 q=0.003900*
AU7	*r=0.086 p=0.264020 q=0.316824*	*r=0.068 p=0.377249 q=0.607253*	*r=0.084 p=0.276855 q=0.825577*
AU9	***r=0.211 p=0.005658 q=0.011316***	***r=0.218 p=0.004265 q=0.002506***	r=0.323 p=0.000016 q=0.000096*
AU10	***r=0.171 p=0.025082 q=0.041043***	*r=0.106 p=0.166199 q=0.226953*	r=0.280 p=0.000210 q=0.000945*
AU12	*r=0.123 p=0.108243 q=0.139170*	***r=0.236 p=0.001924 q=0.011262***	r=0.367 p=0.000001 q=0.000018*
AU14	*r=0.126 p=0.100945 q=0.139170*	*r=0.136 p=0.076349 q=0.596736*	r=0.257 p=0.000696 q=0.002506*
AU15	r=0.327 p=0.000012 q=0.000150*	***r=0.241 p=0.001521 q=0.007627***	r=0.333 p=0.000008 q=0.000072*
AU17	*r=0.081 p=0.293112 q=0.329751*	r=0.311 p=0.000035 q=0.000945*	*r=-0.048 p=0.530432 q=0.607253*
AU20	***r=0.208 p=0.006401 q=0.011522***	***r=0.166 p=0.030229 q=0.037343***	***r=0.244 p=0.001300 q=0.003900***
AU23	*r=0.011 p=0.886410 q=0.926643*	***r=0.222 p=0.003485 q=0.002506***	*r=0.017 p=0.825577 q=0.825577*
AU25	r=0.259 p=0.000619 q=0.001592*	r=0.308 p=0.000042 q=0.000945*	*r=0.043 p=0.573517 q=0.607253*
AU26	***r=0.271 p=0.000334 q=0.001202***	r=0.256 p=0.000713 q=0.007627*	***r=0.177 p=0.020746 q=0.037343***
AU28	***r=0.260 p=0.000585 q=0.001592***	*n/a*	***r=0.226 p=0.002966 q=0.009758***
AU45	r=0.316 p=0.000025 q=0.000150*	*r=0.095 p=0.216920 q=0.355320*	***r=0.217 p=0.004337 q=0.011262***

Results in bold show significant results after FDR (Benjamini-Hochsberg correction, reported as q-values).

*indicates the significant after Bonferroni correction for multiple comparisons (53 tests: αBonferroni=0.05/53≈0.000943), i.e. significance level is p<0.000943.

**Figure 4:**
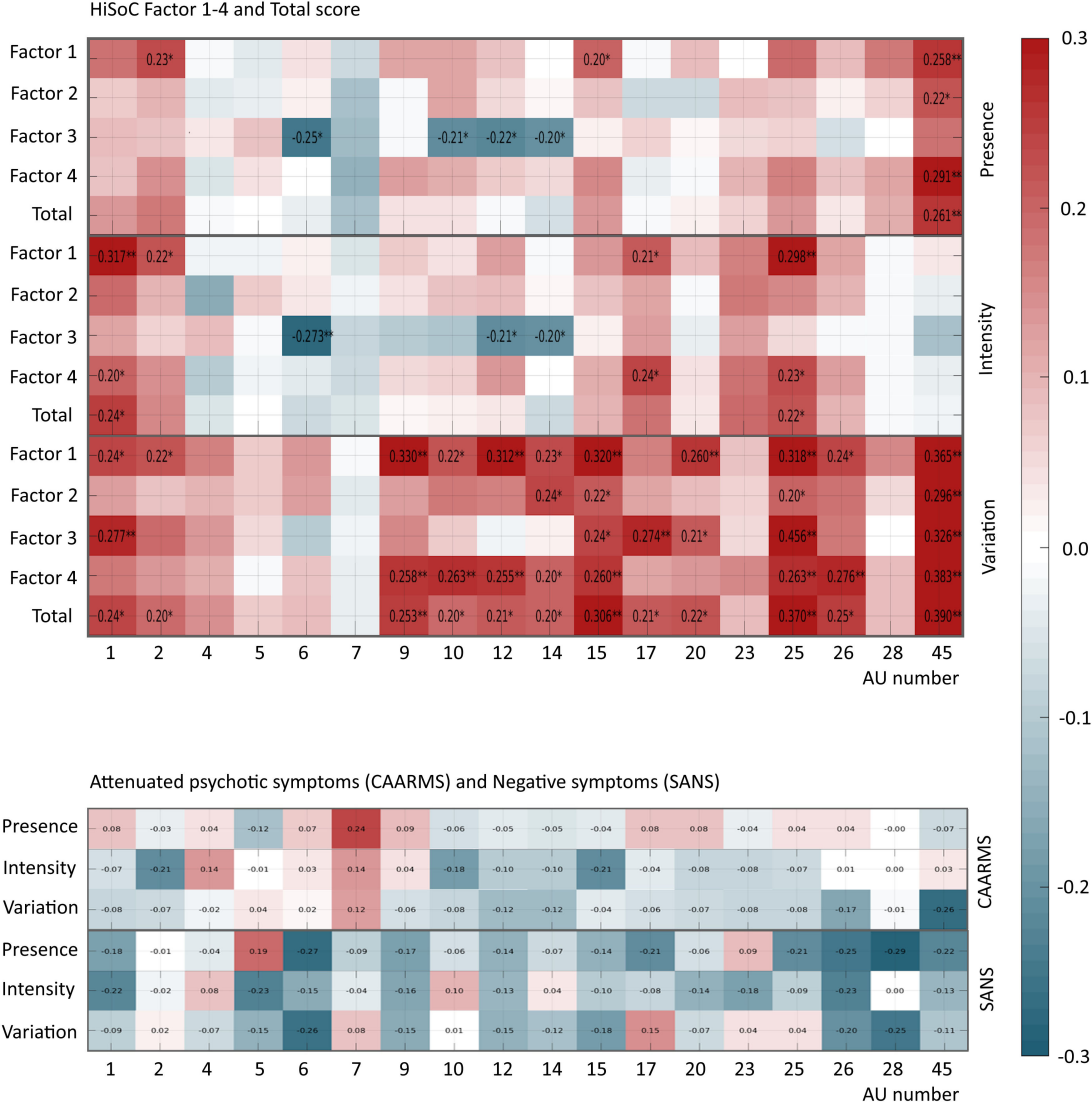
Correlations between action units, social skills, and clinical symptoms. Top Panel illustrates the correlation matrix of Presence, Intensity, and Variation of the AUs with HiSoC Factors. The value in each box is Pearsons correlation coefficient r, indicated only for the significant correlations, omitting non-significant correlations. Blue tones indicate negative correlations, while red tones indicate positive correlations. The significance level p is marked as * when p<0.01, and ** when p<0.001. Bottom panel illustrates the correlation matrix of Presence, Intensity, and Variation (rows) of the 18 AUs (columns) for the clinical measures of CAARMS and SANS. A detailed report of all the correlations coefficients and significance levels are reported in **Supplementary Table 8**. AU, Action Units; CAARMS, the Comprehensive Assessment of At-Risk Mental States; HiSoC, High-Risk Social Challenge; SANS, Scale for the Assessment of Negative Symptoms.

*Post-hoc* analyses revealed significant correlations (all *p*-values<0.001) between Presence, Intensity, and Variation of AUs and HiSoC Factors (Affect, Social-Interpersonal, Odd Behavior & Language, and General impression, and Total score, **Figure 4**). Notably Variation of AU45 (Blink) correlated positively with all HiSoC Factors (*r* = 0.296>0.390).

Within UHR-individuals, attenuated psychotic symptoms (CAARMS score) correlated negatively with Variation AU45 (Blink) (*r* = -0.279, *p* < 0.001). At trend level (*p* = 0.007), CAARMS correlated positively with Presence AU7 (Lid tightener), and SANS negatively (*p* = 0.006) with Presence AU26 (Jaw drop). (**Figure 4**; **Supplementary Table S8**).

*Post-hoc* sensitivity tests comparing AP-naive with AP-exposed UHR-individuals found significant differences on 4 out of the 19 AUs: AU5 (Upper lid raiser), AU15 (Lip corner depressor), AU23 (Lip tightener), and AU26 (Jaw drop). AP-naive UHR-individuals had higher values of presence, intensity, and variation compared to AP-exposed, but only significant differences between AP-exposed UHR-individuals and HC on AU23 and AU26, see **Supplementary Figure S4**, **Supplementary Table S6**.

## 4 Discussion

This study demonstrated statistically significant, albeit modest, classification accuracy in distinguishing UHR-individuals from HC using AU-based machine learning. Although a classification accuracy around 0.7 commonly is considered acceptable, lower accuracies can still provide valuable insights when rigorous validation procedures such as cross-validation and permutation testing are applied (35). The observed sensitivity of 0.76 indicates that the AU-based classifier identifies UHR-individuals reasonably well. In contrast, the low specificity of 0.40 reflects that the model frequently misclassifies misclassify HC as UHR, resulting in a high rate of false positives. These results suggest that AUs carries subtle discriminative information, but there appears to be a substantial overlap in the facial features characterizing UHR-individuals and HC.

Recent classification studies, such as Loch et al. (2023) (36), have reported considerably stronger performance using different machine learning approaches. In their population-based sample of 58 non-help-seeking, medication-naïve individuals with at-risk mental states for psychosis and 70 healthy controls, they achieved an F1-score of 83%, accuracy of 85%, and an AUC of 93%. One possible explanation for this notably strong result may be the extensive set of extracted facial features (649), which included a wide range of metrics beyond AUs, However, the combination of unusually high performance with a high-dimensional feature space relative to a moderate sample size raises concerns about potential overfitting. Moreover, a population-based sample of non-help seeking at-risk individuals is likely to be more homogeneous than help-seeking UHR individuals, who commonly present with greater clinical complexity, including partial medication use and higher rates of comorbidity. As a result, Loch et al. (2023) (36) may have worked with data with less noise and a clearer separation between at-risk and controls, despite the modest sample size.

Interestingly, our *post-hoc* sensitivity analyses suggest that antipsychotic (AP)-naïve UHR-individuals exhibit AU-features more comparable to HC, whereas AP-exposed UHR-individuals show significantly lower presence, intensity and variation in specific AUs. This implies that the subtle group-level abnormalities in UHR facial expressivity may mask more pronounced deficits within specific subgroups, as reflected by those prescribed AP-medication. However, we cannot determine whether these subgroup differences are attributable to the effects of medication, or whether they reflect more underlying illness severity among AP-exposed UHR-individuals. We found no demographic or clinical differences between the UHR-subgroups, and only a limited number of studies have directly investigated the potential impact of AP-medication on AUs in patients with established schizophrenia. Tron et al. (2015) (37) explicitly noted in their study of fully medicated patients with schizophrenia that neuroleptic treatment likely contributes to reduced facial activity. Similarly, Schneider et al. (1992) reported a marked reduction in facial expressivity among initially AP-naive patients with schizophrenia spectrum disorders after three weeks of medication, whereas those patients already medicated at baseline showed persistently low levels of facial expressivity. More recently, Ambrosen et al. (2025) (38) examined initially AP-naive patients with first episode psychosis, and reported that pre-treatment AU-patterns predicted subsequent response to antipsychotic medication. This finding underscores a robust link between AUs and antipsychotic effects. To date, however, no studies have investigated the potential impact of AP-medication on AU-features in UHR-individuals, leaving this an important open question for future research.

Our findings expand the limited research of facial emotion expression deficits in UHR-individuals by providing a higher-resolution characterization of facial behavior using AUs. Most previous studies such as Gupta et al. (14, 23) have primarily examined higher-level emotional categories (e.g. happy, sad, anger, fear) (22). These studies typically report reduced joy among UHR-individuals using automated coding approaches, findings that correlate with clinician-rated blunted affect. While this work has been valuable in establishing broad emotional abnormalities, it does not directly quantify the underlying facial muscular movements that give rise to these expressions. By focusing on moment-to-moment muscular activation rather than discrete emotional categories, our findings align with contemporary models of emotion that emphasize dimensional and process-based perspectives rather than static categories. By linking granular AU-level activity to clinical assessments, we offer a more mechanistic and finer-grained understanding of facial expressivity in UHR individuals. This approach may be especially relevant for UHR-populations, where subtle signs of psychopathology are easily overlooked (14). This challenge was also highlighted in Trémeau et al. (2005) (10), who reported no group differences between schizophrenia and depression when using manually coded FACS, suggesting that low-level abnormalities may be too subtle to detect without automated, high-sensitivity methods. Capturing these subtle alterations is important because facial expressivity is closely linked to social functioning, treatment responsiveness, and illness trajectory in psychosis-spectrum disorders. Therefore, AU-based methods may serve as a more sensitive marker of emerging psychopathology than categorical emotion labels alone.

Strong correlations between clinician-rated facial affect (HiSoC item 1) and the AU metrics, together with multiple positive correlations across the broader the HiSoC factors and clinical symptoms, align with prior studies validating automated coding against manual FACS scoring (9). Our findings support hypothesis that basic anatomical impairments in facial expression impairments among UHR-individuals are linked to reduced social skills (24).

Nevertheless, our results should be interpreted with some caution when relying on automated FACS outputs, particularly in clinical populations, given the limited validation to date (18, 19). Importantly, we do observe meaningful associations between AUs and clinical symptom severity in the UHR-individuals, both attenuated psychotic symptoms (CAARMS) and negative symptoms (SANS), which strengthens the clinical validity of automated AU outputs beyond mere classification. Taken together, our findings suggest that automated AU has promising potential as a clinically validated and relevant tool for measuring emotion-processing deficits in UHR-populations, supporting a broader utility of computational methods in clinical settings. Because the approach is potentially applicable to mobile devices (35), it may be especially feasible for implementation in everyday psychiatric practice. By demonstrating strong alignment between automated coding and clinician ratings of facial expressivity, our findings are particularly encouraging for applications in low-resource clinical settings.

The subtle abnormalities in facial emotion expression observed in UHR-individuals, particularly the increased Presence of AU7 (lid tightener), has frequently been associated with anxiety and mood disorders (39), both of which are highly prevalent in UHR-populations (40). Although the correlation between AU7 and attenuated psychotic symptoms (CAARMS) did not remain significant after correction for multiple comparisons, the moderate effect size suggests that AU7 may carry clinical relevance clinical relevance beyond mood symptoms and may reflect features specifically linked to UHR-status.

We further observed less Variation in AU45 (blinking), indicating reduced facial expression variability in UHR-individuals. This finding aligns with evidence demonstrating that HCs typically show a wider range of facial expressions across both positive and negative emotions (41), whereas reduced variability may reflect emotional blunting, a core negative symptom in psychotic disorders (42). The association between AU45 (blinking) and attenuated psychotic symptoms is particularly noteworthy. Elevated blink rates have been observed in unmedicated patients with schizophrenia compared to healthy (43) controls (44), and spontaneous blink rate has been proposed as an indirect measure of central dopaminergic function. Prior studies have linked blink rate to both positive (45) and negative symptoms, neuroleptic dosage (46) and treatment response (45).

The central role of dopaminergic system in the treatment of schizophrenia, particularly through the dopamine blocking effects of antipsychotic medication, spontaneous blink rate may represent a potentially informative clinical marker. However, the evidence remains inconsistent. Some studies in healthy individuals report no (47) or even negative associations (43) between blink rate and dopamine activity; underscoring a need for interpretive caution. Although we did not observe any effect of antipsychotic medication in our sample, the study was not specifically designed to evaluate medication influences. Consequently, our findings linking blinking to attenuated psychotic symptoms but not antipsychotic medication status, should be viewed as hypothesis generating and warrant future studies.

A key strength of this study is the novel application of automated analysis of discrete AUs in a comparatively large UHR sample. Despite a smaller HC sample, several AUs nevertheless emerged as potential screening markers, though replication in a larger balanced samples remains essential. Importantly, the enriched dataset allowed for nuanced examinations of associations between granulated AUs and diverse clinical measures, thereby contributing meaningfully to the growing clinical validation of automated facial-expression tools.

This study also has limitations. The structured HiSoC tasks may constrain naturalistic facial behavior and reduce temporal variability in expressions (33). However, the subtle alterations of facial expression among UHR-individuals may become more apparent only under conditions of emotional arousal (14), supporting the relevance of the task design (24). Additionally, while brief video segments may restrict generalizability, research on “thin slicing” demonstrates considerable prediction accuracy comparable to longer recordings (48). Nevertheless, future work should integrate more ecologically valid paradigms to complement structured assessments.

In conclusion, automated facial coding represents a clinically promising and scalable method for detecting subtle emotion-expression impairments in UHR-individuals. By enabling fine-grained measurement of facial features, these tools hold potential for early identification, refined assessment of symptom dimensions, and support in guiding targeted interventions. Continued validation in larger and more diverse clinical samples will be critical for advancing their integration into routine clinical practice.

## Data Availability

The datasets generated and/or analyzed during the current study are available from the corresponding author upon reasonable request. Requests to access these datasets should be directed to tina.dam.kristensen@regionh.dk.
