# Supplementary material for: Subtle impairments of facial emotion expressions in individuals at ultra-high risk for psychosis
Source: Front Psychiatry. 2026 Apr 1;17:1743609. doi: 10.3389/fpsyt.2026.1743609 (PMC13079383; doi:10.3389/fpsyt.2026.1743609)
Supplement: Supplementary file 1 [file Supplementaryfile1.docx]

Supplementary Figure S1


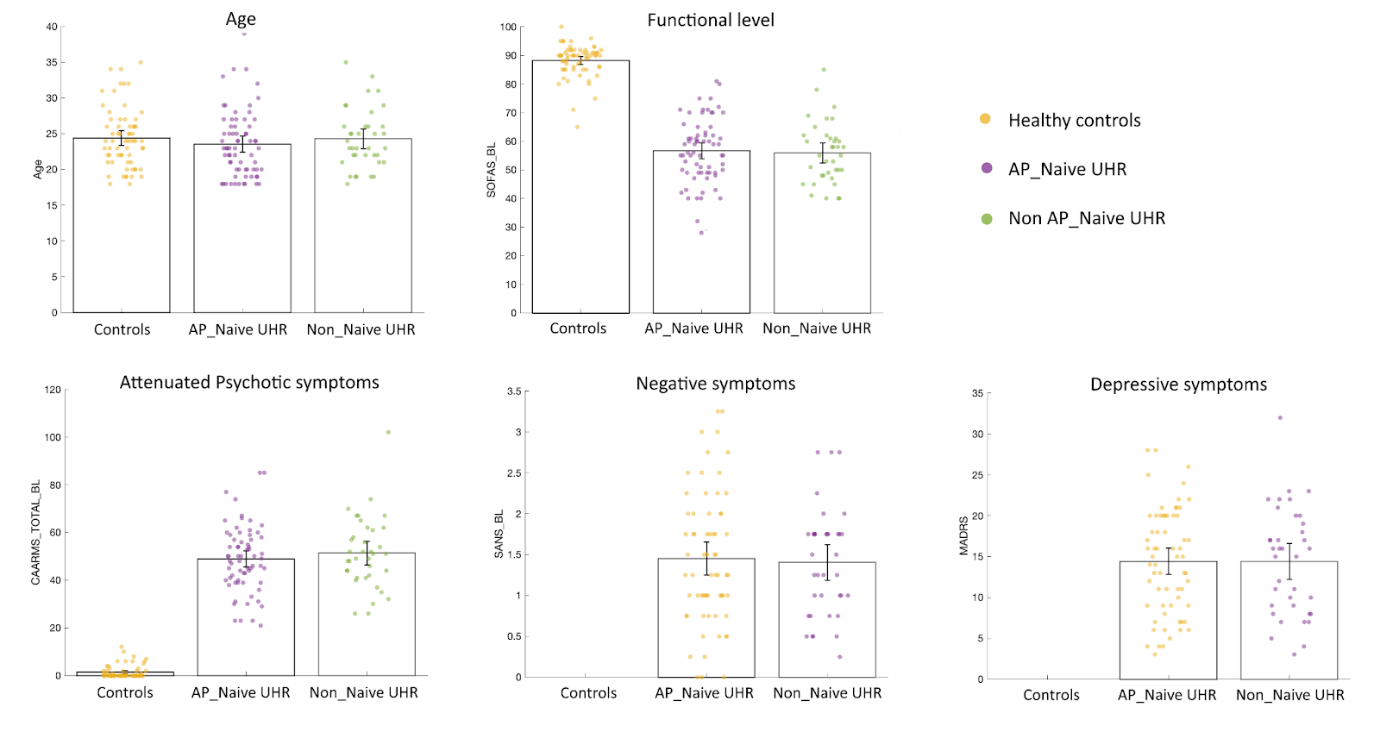


*Figure S1 illustrates the distribution of demographic and clinical variables when comparing AP-naive with AP-exposed UHR-individuals, and both groups with healthy controls (when optional). As also reported in Supplementary Table Sx., AP-naive do not differ from AP-exposed UHR-individuals on any clinical or demographic variables.*

*Functional levels: as measured by SOFAS; Attenuated psychotic symptoms as measured by CAARMS; Negative symptoms as measured by SANS; and Depressive symptoms as measured by MADRS.*

**Figure S2. Group difference of Action Units**


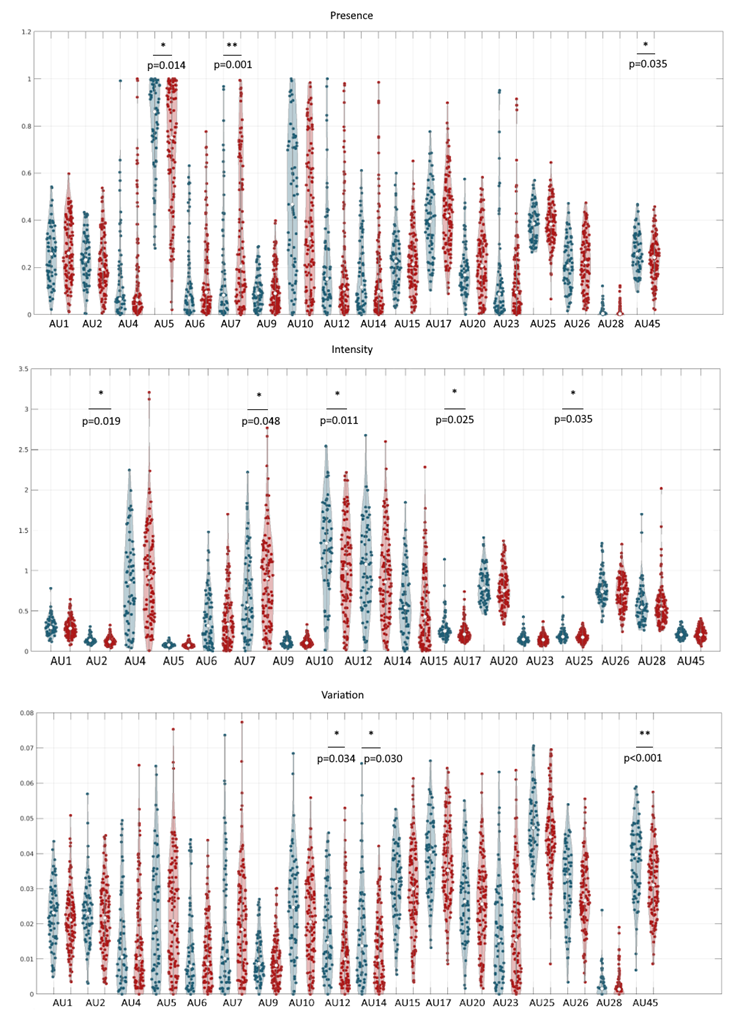


*Figure S2 illustrates the results from the univariate tests of Action Unit group differences comparing UHR-individuals (red dots) with healthy controls (blue dots). In top panel violinplots illustrate the distribution of participants for Presence of AUs, middle panel illustrate the groupwise distribution of AU Intensity; and the bottom panel the groupwise distribution of Variation. Significance is marked with *p<0.05; **Significant after Bonferroni correction*

**Supplementary Figure S4 Significant differences in Action Units comparing antipsychotic-naïve with antipsychotic-exposed individuals at ultra-high risk for psychosis**


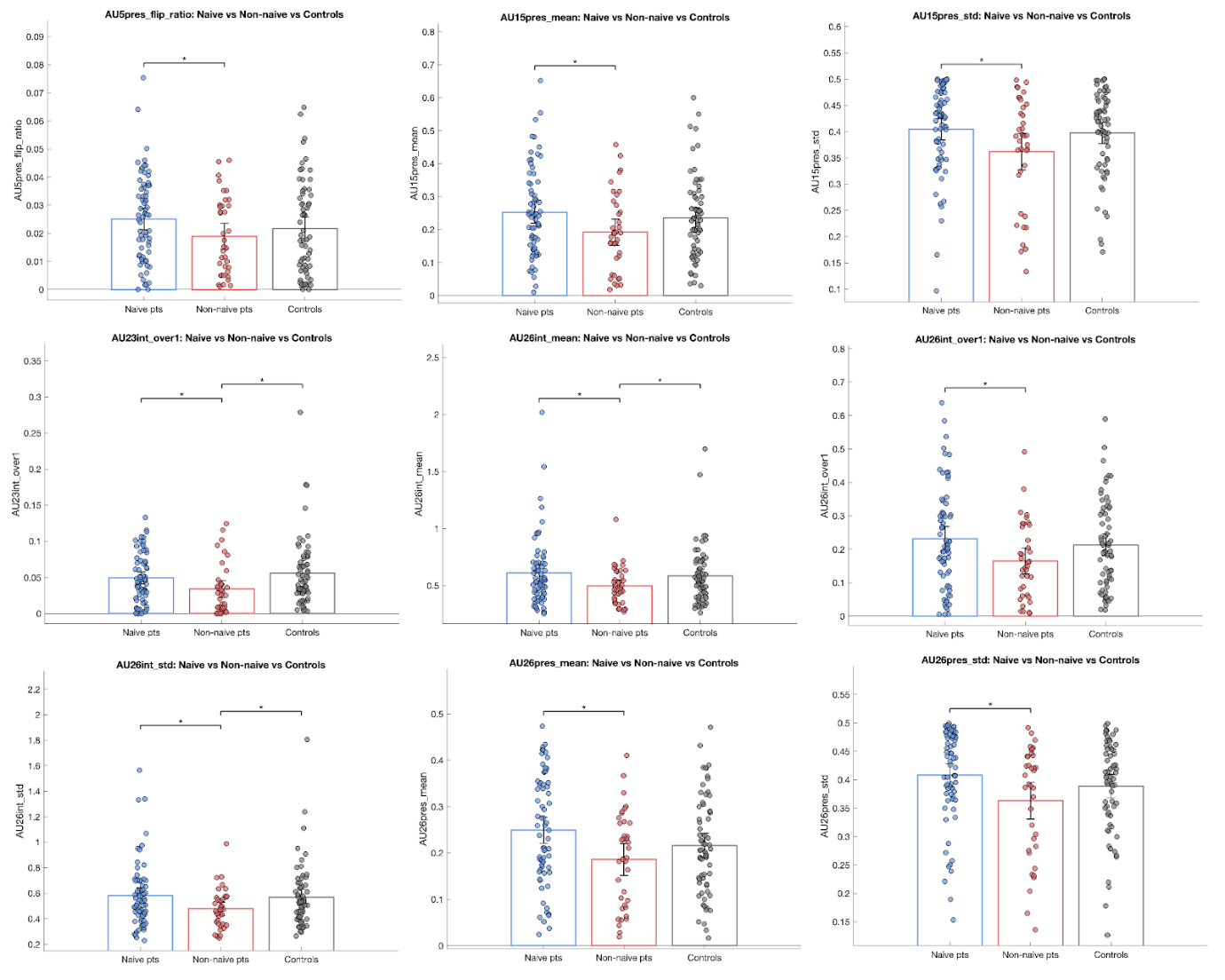


*Supplementary Figure S3 illustrate the distribution of the AU variables which differed significantly when comparing AP-naive with AP-exposed UHR-individuals, and both groups with healthy controls. As also reported in Supplementary Table S6, AP-naive differ from AP-exposed UHR-individuals on measures form 4 out of the 19 AUs: AU 5, AU 15, AU 23, and AU 26, on all measures with higher values to AP-naive UHR-individuals. It visually appears as the AP-naive UHR-individuals to a higher degree attenuate the AU-levels of HC, although we only find significant differences between AP-exposed UHR and HC on AU 23 and AU 26.*

**Supplementary Table S1 Rating of HiSoC**

| FACTOR | ITEM |
| --- | --- |
| AFFECT | 1. Facial affect. The degree, amount, range, and naturalness of emotions displayed via *facial* expression. Examples: dull facial expression, infrequent blinking, rarely if ever smiling. |
|  | 2. Non-verbal expression. The extend of gestures used for communicating meaning, amount, range, naturalness of emotions displayed via *body* language. |
|  | 3. Appropriate affect. Communication of emotions through facial expression, gestures, and vocal tone – rating of the appropriateness of the emotions. |
|  | 4. Verbal expression. Language and tone – amount, range, and naturalness of tonal inflections. |
|  | 5. Gaze. Frequency, duration, appropriateness, and naturalness of eye contact. |
|  | 6. Physical anergia. Energy level and muscular tonus. |
| ODD BEHAVIOR AND LANGUAGE | 7. Appearance, unusual or odd behavior, such as unusual gestures with face and hands, inappropriate dressing, awkward ticks or jerks. |
|  | 8. Content. Unusual or odd speech, such as irrelevant answer, verbigeration, neologisms, word salat, echolalia, fusion. |
|  | 9. Tangential speech. Tangentiality, derailment, loose associations, flight of ideas. |
|  | 10. Speech content valence. Positive and negative valence of the speech. |
|  | 11. Clear communication. Unclarity from: missing references, ambiguous words, poor grammar. |
| SOCIAL-INTERPERSONAL | 12. Fluency of speech. Stuttering, pauses, use of “um”. Impact on fluency and effectiveness of communication. |
|  | 13. Guardedness. The extend the participant seems unwilling to share information. |
|  | 14. Social anxiety. Amount of anxiety displayed (stuttering, fidgeting, shaking, etc. and the impact the anxiety has on the speech. |
|  | 15. Engagement. Level of interest in the task. |
| OVERALL RATING | 16. Overall performance and ability to communicate in an effective and meaningful way. |

| AU | 1 | 2 | 4 | 5 | 6 | 7 | 9 | 10 | 12 | 14 | 15 | 17 | 20 | 23 | 25 | 26 | 45 |
| --- | --- | --- | --- | --- | --- | --- | --- | --- | --- | --- | --- | --- | --- | --- | --- | --- | --- |
| x | -0.052 | 0.033 | -0.065 | -0.030 | -0.009 | -0.025 | 0.063 | 0.099 | 0.078 | 0.245 | 0.011 | 0.041 | 0.068 | 0.080 | -0.069 | -0.106 | -0.023 |
| y | 0.095 | 0.137 | 0.268 | 0.083 | -0.251 | -0.121 | 0.145 | -0.076 | -0.044 | 0.010 | -0.047 | 0.174 | -0.023 | 0.200 | 0.071 | 0.113 | -0.001 |
| z | -0.103 | -0.121 | -0.297 | 0.122 | 0.046 | 0.054 | -0.057 | 0.139 | -0.028 | 0.171 | -0.069 | -0.050 | -0.148 | 0.027 | -0.007 | -0.053 | 0.022 |

**Table S2. Correlations between Action Units and head pose pitch (x), yaw (y), and roll (z)**

Table S2 report the correlation coefficients between Action Units and head pose defined as pitch (x), yaw (y), and roll (Z).

**Supplementary Table S3 Comparing antipsychotic-naïve, antipsychotic-exposed individuals at ultrahigh risk for psychosis, and healthy controls on demographic and clinical data.**

| Variable | HC (N=65) | AP naive UHR (N=68) | Non naive UHR (N=37) | F-value ANOVA | p-value ANOVA | p-value Naive vs HC | p-value NonNaive vs HC | p-value AP naive vs Non Naive UHR |
| --- | --- | --- | --- | --- | --- | --- | --- | --- |
| Age | 24.38 (4.18) | 23.51 (4.60) | 24.30 (4.16) | 0.7627 | 0.468 | 0.578 | 0.919 | 0.378 |
| Functional level | 88.28 (5.69) | 56.69 (11.17) | 55.89 (10.63) | 234.51 | <0.001 | <0.001 | <0.001 | 0.721 |
| Attenuated psychotic symptoms (CAARMS) | 1.46 (2.69) | 48.94 (13.84) | 51.41 (14.92) | 368.05 | <0.001 | na | na | 0.411 |
| Negative symptoms (SANS) | NA | 1.45 (0.81) | 1.41 (0.65) | 0.0789 | na | na | na | 0.767 |
| Sex | 27 (41.5%) | 31 (45.6%) | 18 (48.6%) | na | na | 0.1900 | 0.537 | 0.839 |

**Supplementary Table S4 Univariate group differences on Action Units comparing individuals at ultra-high risk for psychosis with healthy controls.**

| **Presence** | **Intensity** | **Variation** |
| --- | --- | --- |
| AU01: significant 0, p-value 0.310997  AU02: significant 0, p-value 0.363420  AU04: significant 0, p-value 0.573907  **AU05: significant 1, p-value 0.014593**  AU06: significant 0, p-value 0.583739  **AU07: significant 1, p-value 0.001203***  AU09: significant 0, p-value 0.312909  AU10: significant 0, p-value 0.087651  AU12: significant 0, p-value 0.550316  AU14: significant 0, p-value 0.946965  AU15: significant 0, p-value 0.824675  AU17: significant 0, p-value 0.187869  AU20: significant 0, p-value 0.127550  AU23: significant 0, p-value 0.833120  AU25: significant 0, p-value 0.930109  AU26: significant 0, p-value 0.549986  AU28: significant 0, p-value 0.846413  **AU45: significant 1, p-value 0.035327** | AU01: significant 0, p-value 0.371891  AU02: significant 0, p-value 0.057706  AU04: significant 0, p-value 0.415306  AU05: significant 0, p-value 0.619244  AU06: significant 0, p-value 0.976761  **AU07: significant 1, p-value 0.037189**  AU09: significant 0, p-value 0.936143  **AU10: significant 1, p-value 0.014488**  **AU12: significant 1, p-value 0.042143**  AU14: significant 0, p-value 0.280839  AU15: significant 0, p-value 0.090838  AU17: significant 0, p-value 0.193470  AU20: significant 0, p-value 0.382916  AU23: significant 0, p-value 0.063564  AU25: significant 0, p-value 0.198214  AU26: significant 0, p-value 0.730460  AU28. na  AU45: significant 0, p-value 0.137532 | AU01: significant 0, p-value 0.396173  AU02: significant 0, p-value 0.385422  AU04: significant 0, p-value 0.996591  AU05: significant 0, p-value 0.617668  AU06: significant 0, p-value 0.701222  AU07: significant 0, p-value 0.376366  AU09: significant 0, p-value 0.155815  AU10: significant 0, p-value 0.092064  **AU12: significant 1, p-value 0.034912**  **AU14: significant 1, p-value 0.030409**  AU15: significant 0, p-value 0.491576  AU17: significant 0, p-value 0.208888  AU20: significant 0, p-value 0.359026  AU23: significant 0, p-value 0.920694  AU25: significant 0, p-value 0.095787  AU26: significant 0, p-value 0.060714  AU28: significant 0, p-value 0.744022  **AU45: significant 1, p-value 0.000107*** |

Bold: significant at raw p-value; * Significant after FDR correction

**Table S5 Sex as covariate when comparing individuals at ultra-high risk for psychosis with healthy controls on Presence AU7 and Variation AU45**

| Measure | P value Group ttest | FDR P value Group ttest | Cohens d | P value Group with Sex as covariate | FDR p value Group with Sex as covariate |
| --- | --- | --- | --- | --- | --- |
| Presence AU7 | 0.0007 | 0.0141 | 0.5137 | 0.0015 | 0.0275 |
| Variation AU45 | 0.0001 | 0.0020 | -0.6369 | 9.42712E-05 | 0.0016 |

**Supplementary Table S6 Action Units with significant effect of antipsychotic medication**

| Measure | Mean Naive | Mean Exposed | Mean HC | t_test Naive vs Exposed | P Naive vs Exposed | t_test Naive vs HC | P Naive vs HC | t_test Exposed vs HC | P Exposed vs HC |
| --- | --- | --- | --- | --- | --- | --- | --- | --- | --- |
| AU26int_mean | 0,6121 | 0,4987 | 0,5856 | 2,5204 | 0,0132 | 0,5564 | 0,5788 | -2,1222 | 0,0363 |
| AU26int_std | 0,5808 | 0,4796 | 0,5693 | 2,5257 | 0,0130 | 0,2645 | 0,7917 | -2,2544 | 0,02636 |
| AU23int_over1 | 0,0492 | 0,0339 | 0,0560 | 2,1518 | 0,0347 | -0,9432 | 0,3475 | -2,6955 | 0,0083 |
| AU26int_over1 | 0,2311 | 0,1646 | 0,2132 | 2,5132 | 0,0136 | 0,7247 | 0,4699 | -1,9458 | 0,0550 |
| AU15pres_mean | 0,2525 | 0,1923 | 0,2354 | 2,3681 | 0,0202 | 0,7567 | 0,4505 | -1,7281 | 0,0878 |
| AU26pres_mean | 0,2491 | 0,1860 | 0,2159 | 2,8516 | 0,0054 | 1,7129 | 0,0891 | -1,3933 | 0,1675 |
| AU15pres_std | 0,4046 | 0,3619 | 0,3972 | 2,1275 | 0,0373 | 0,5086 | 0,6118 | -1,7682 | 0,0820 |
| AU26pres_std | 0,4081 | 0,3631 | 0,3891 | 2,3750 | 0,0205 | 1,3173 | 0,1900 | -1,3619 | 0,1778 |
| AU5pres_flip_ratio | 0,0250 | 0,01897 | 0,02170 | 2,0522 | 0,0432 | 1,1903 | 0,2360 | -0,8909 | 0,3754 |

**Supplementary Table S7 Correlation between HiSoC Item 1 and Action Units in healthy controls and individuals at ultra-high risk for psychosis**

|  | **HC** | | | **UHR** | | |
| --- | --- | --- | --- | --- | --- | --- |
|  | **Presence** | **Intensity** | **Variability** | **Presence** | **Intensity** | **Variability** |
| **AU1** | **r=0.327 p=0.007929 q=0.023787** | **r=0.578 p<0.000001 q<0.000001** | r=0.095 p=0.452201 q=0.755542 | **r=0.457 p=0.000001 q=0.000018 *** | **r=0.424 p=0.000006 q=0.000102 *** | **r=0.155 p=0.112202 q=0.183603** |
| **AU2** | **r=0.455 p=0.000141 q=0.002538** | r=0.311 p=0.011621 q=0.065852 | r=0.014 p=0.911791 q=0.965426 | r=0.196 p=0.043822 q=0.060677 | **r=0.243 p=0.011972 q=0.020352** | r=0.114 p=0.242724 q=0.336079 |
| **AU4** | r=0.139 p=0.270646 q=0.405969 | r=0.023 p=0.856354 q=0.909876 | r=0.081 p=0.519354 q=0.755542 | r=-0.037 p=0.708495 q=0.750171 | r=0.011 p=0.910449 q=0.910449 | r=0.156 p=0.110699 q=0.183603 |
| **AU5** | **r=-0.369 p=0.002468 q=0.015828** | r=0.182 p=0.145875 q=0.314298 | r=0.232 p=0.062961 q=0.355320 | **r=-0.245 p=0.011305 q=0.018499** | r=0.143 p=0.143417 q=0.174149 | r=0.100 p=0.306790 q=0.351179 |
| **AU6** | r=0.119 p=0.343561 q=0.475700 | r=0.075 p=0.552991 q=0.862304 | r=0.121 p=0.337878 q=0.675756 | **r=0.352 p=0.000216 q=0.000972** | **r=0.331 p=0.000521 q=0.002605** | **r=0.302 p=0.001648 q=0.004944** |
| **AU7** | r=0.266 p=0.032165 q=0.065696 | r=0.201 p=0.109235 q=0.314298 | r=0.213 p=0.088557 q=0.355320 | **r=0.229 p=0.018204 q=0.027306** | r=0.162 p=0.098017 q=0.128176 | r=0.099 p=0.312159 q=0.351179 |
| **AU9** | **r=0.367 p=0.002638 q=0.015828** | r=0.335 p=0.006411 q=0.054494 | r=0.308 p=0.012578 q=0.226404 | **r=0.250 p=0.009789 q=0.017620** | **r=0.217 p=0.025299 q=0.035840** | **r=0.306 p=0.001433 q=0.004944** |
| **AU10** | r=0.078 p=0.538265 q=0.692055 | r=-0.055 p=0.661760 q=0.865378 | r=0.088 p=0.487631 q=0.755542 | r=0.154 p=0.115705 q=0.143029 | r=0.055 p=0.573293 q=0.609124 | **r=0.342 p=0.000336 q=0.002016 *** |
| **AU12** | r=0.049 p=0.695891 q=0.725726 | r=0.024 p=0.849112 q=0.909876 | r=0.273 p=0.027758 q=0.249822 | r=0.152 p=0.119191 q=0.143029 | **r=0.273 p=0.004592 q=0.013011** | **r=0.368 p=0.000104 q=0.000936 *** |
| **AU14** | r=0.169 p=0.177487 q=0.290433 | r=0.065 p=0.608685 q=0.862304 | r=0.135 p=0.282088 q=0.634698 | r=0.143 p=0.144783 q=0.162881 | r=0.138 p=0.159145 q=0.180364 | **r=0.254 p=0.008523 q=0.019177** |
| **AU15** | r=0.289 p=0.019763 q=0.050819 | r=0.107 p=0.395075 q=0.746253 | r=0.198 p=0.114539 q=0.355320 | **r=0.407 p=0.000015 q=0.000135 *** | **r=0.264 p=0.006197 q=0.015050** | **r=0.422 p=0.000006 q=0.000108 *** |
| **AU17** | r=-0.044 p=0.725726 q=0.725726 | r=0.185 p=0.140918 q=0.314298 | r=-0.075 p=0.555049 q=0.755542 | **r=0.253 p=0.008999 q=0.017620** | **r=0.360 p=0.000149 q=0.001266** | r=-0.125 p=0.200029 q=0.300044 |
| **AU20** | r=0.206 p=0.099696 q=0.179453 | r=-0.007 p=0.955552 q=0.955552 | r=0.056 p=0.658964 q=0.755542 | **r=0.341 p=0.000346 q=0.001038** | **r=0.234 p=0.015856 q=0.024505** | **r=0.333 p=0.000496 q=0.002232 *** |
| **AU23** | r=0.054 p=0.669503 q=0.725726 | r=0.069 p=0.585770 q=0.862304 | r=0.067 p=0.598099 q=0.755542 | r=-0.030 p=0.757449 q=0.757449 | **r=0.254 p=0.008735 q=0.018060** | r=-0.010 p=0.916392 q=0.916392 |
| **AU25** | r=0.068 p=0.591011 q=0.709213 | r=0.272 p=0.028245 q=0.120041 | r=0.002 p=0.984849 q=0.984849 | **r=0.399 p=0.000023 q=0.000138 *** | **r=0.320 p=0.000823 q=0.002798** | r=-0.029 p=0.765659 q=0.810698 |
| **AU26** | **r=0.329 p=0.007475 q=0.023787** | r=0.182 p=0.147905 q=0.314298 | r=0.159 p=0.206925 q=0.532093 | **r=0.343 p=0.000314 q=0.001038** | **r=0.327 p=0.000613 q=0.002605** | r=0.103 p=0.294889 q=0.351179 |
| **AU28** | r=0.265 p=0.032848 q=0.065696 | na | r=0.196 p=0.118440 q=0.355320 | **r=0.303 p=0.001578 q=0.004058** | **na** | **r=0.274 p=0.004452 q=0.011448** |
| **AU45** | **r=0.329 p=0.007368 q=0.023787** | r=-0.040 p=0.749365 q=0.909876 | r=-0.054 p=0.671593 q=0.755542 | **r=0.254 p=0.008564 q=0.017620** | **r=0.251 p=0.009561 q=0.018060** | r=0.167 p=0.087720 q=0.175440 |

**Table S8. Correlations between Presence, Intensity, and Variation of the 18 Action Units and clinical measures in individuals at ultra-high risk of psychosis**

|  | **CAARMS** | | | **SANS** | | |
| --- | --- | --- | --- | --- | --- | --- |
|  | Intensity | Presence | Variability | Intensity | Presence | Variability |
| **AU 01** | r=-0.060 p=0.438 | r=0.089 p=0.250 | r=-0.075 p=0.330 | **r=-0.233 p=0.019** | r=-0.185 p=0.062 | r=-0.070 p=0.486 |
| **AU 02** | **r=-0.161 p=0.036** | r=-0.031 p=0.692 | r=-0.107 p=0.164 | r=-0.082 p=0.415 | r=-0.016 p=0.871 | r=0.025 p=0.804 |
| **AU 04** | r=0.114 p=0.138 | r=0.063 p=0.416 | r=-0.001 p=0.990 | r=0.136 p=0.173 | r=0.041 p=0.681 | r=-0.052 p=0.605 |
| **AU 05** | r=0.044 p=0.567 | **r=-0.161 p=0.036** | r=0.039 p=0.616 | r=-0.128 p=0.199 | r=0.143 p=0.151 | r=-0.087 p=0.383 |
| **AU 06** | r=0.000 p=0.997 | r=0.053 p=0.495 | r=-0.018 p=0.815 | r=-0.099 p=0.321 | **r=-0.200 p=0.044** | **r=-0.224 p=0.024** |
| **AU 07** | r=0.139 p=0.071 | **r=0.207 p=0.007** | r=0.076 p=0.323 | r=0.007 p=0.946 | r=-0.089 p=0.374 | r=0.116 p=0.246 |
| **AU 09** | r=0.030 p=0.702 | r=0.099 p=0.200 | r=-0.090 p=0.246 | r=-0.078 p=0.433 | r=-0.137 p=0.168 | **r=-0.204 p=0.039** |
| **AU 10** | **r=-0.176 p=0.021** | r=-0.088 p=0.253 | r=-0.121 p=0.117 | r=0.067 p=0.503 | r=-0.072 p=0.469 | r=0.002 p=0.983 |
| **AU 12** | r=-0.130 p=0.090 | r=-0.023 p=0.767 | r=-0.118 p=0.125 | r=-0.125 p=0.210 | r=-0.128 p=0.202 | r=-0.170 p=0.088 |
| **AU 14** | r=-0.084 p=0.278 | r=0.023 p=0.770 | **r=-0.152 p=0.048** | r=0.045 p=0.654 | r=-0.045 p=0.656 | r=-0.182 p=0.067 |
| **AU 15** | r=-0.163 p=0.034 | r=-0.033 p=0.665 | r=-0.054 p=0.483 | r=-0.049 p=0.627 | r=-0.139 p=0.164 | r=-0.192 p=0.053 |
| **AU 17** | r=-0.076 p=0.322 | r=0.106 p=0.170 | r=-0.084 p=0.279 | r=-0.095 p=0.344 | r=-0.193 p=0.052 | r=0.115 p=0.251 |
| **AU 20** | r=-0.077 p=0.318 | r=0.093 p=0.229 | r=-0.083 p=0.283 | r=-0.045 p=0.656 | r=-0.044 p=0.663 | r=-0.093 p=0.354 |
| **AU 23** | r=-0.098 p=0.204 | r=-0.036 p=0.640 | r=-0.046 p=0.553 | r=-0.136 p=0.172 | r=0.101 p=0.310 | r=0.102 p=0.309 |
| **AU 25** | r=-0.076 p=0.324 | r=0.017 p=0.825 | r=-0.109 p=0.156 | r=-0.063 p=0.529 | **r=-0.233 p=0.018** | r=0.002 p=0.987 |
| **AU 26** | r=0.029 p=0.705 | r=0.050 p=0.517 | r=-0.136 p=0.077 | **r=-0.195 p<0.050** | **r=-0.269 p=0.006** | r=-0.142 p=0.155 |
| **AU 45** | r=0.135 p=0.079 | r=-0.032 p=0.679 | **r=-0.279 p<0.001** | r=-0.119 p=0.233 | **r=-0.246 p=0.013** | **r=-0.215 p=0.030** |

**Supplementary Text S1 Repeated Nested Stratified Cross-Validation**

**Feature Extraction and Selection**

We used handcrafted features derived from OpenFace outputs, which included:

- AU intensity (range: 0–5) per frame
- AU presence (binary: 0 or 1) per frame

For each AU, the following features were computed:

- Mean intensity across frames (AU_int_mean)
- Standard deviation of intensity
- Proportion of frames with intensity > 1 (AU_int_over1)
- Mean presence across frames (AU_presence_mean)
- Standard deviation of presence
- Presence flip ratio: frequency of transitions between 0 and 1, normalized by the number of frames (AU_pres_flip_ratio)

Feature selection was conducted within the training data of each outer fold to identify the most informative AUs for classification.

**Permutation Testing**

To assess the statistical significance of the model’s performance, we conducted permutation testing with 100 permutations. For each permutation:

1. The labels in the training set were randomly shuffled.
2. The full nested cross-validation procedure was repeated using the permuted labels in both the inner and outer loops.
3. Feature selection and hyperparameter tuning were performed as in the original setup.
4. The model was trained on the permuted training data and evaluated on the true test labels.
5. Accuracy and AUC were recorded for each permutation.

The p-value was computed by comparing the true model performance to the distribution of performance metrics obtained from the permuted runs:

p=Number of permutations with performance ≥ true performanceTotal number of permutations*p*=Total number of permutationsNumber of permutations with performance ≥ true performance​
